# Therapeutic efficacy of artemether-lumefantrine against uncomplicated *Plasmodium falciparum* malaria in a high-transmission area in northwest Ethiopia

**DOI:** 10.1371/journal.pone.0176004

**Published:** 2017-04-26

**Authors:** Michael Teklemariam, Ashenafi Assefa, Moges Kassa, Hussien Mohammed, Hassen Mamo

**Affiliations:** 1 Department of Microbial, Cellular and Molecular Biology; College of Natural Sciences, Addis Ababa University, Addis Ababa, Ethiopia; 2 Bacterial, Parasitic and Zoonotic Diseases Research Directorate, Ethiopian Public Health Institute, Addis Ababa, Ethiopia; Université Pierre et Marie Curie, FRANCE

## Abstract

Malaria, particularly due to *Plasmodium falciparum*, remains a major public health threat in Ethiopia. Artemether-lumefantine (AL) has been the first-line antimalarial drug against uncomplicated *P*. *falciparum* malaria in the country since 2004. Regular monitoring of antimalarial drugs is recommended by the World Health Organization (WHO) to help early detection of drug resistant strains of the parasite and contain their rapid spread. The objective of this study was to assess the therapeutic efficacy of AL in a high-transmission setting in Ethiopia. The study site was Setit Humera, northwest Ethiopia. Single-arm prospective study of a 28-day follow-up was conducted from October 2014 to January 2015 according to the revised WHO 2009 drug efficacy study protocol. Study end-points were classified into primary end-point and secondary end-point. While the primary end-point was the day-28 adequate clinical and parasitological response the secondary end-points were clinical and parasitological evaluations (parasite, fever and gametocyte clearance rate, incidence of drug adverse events) and the relative increment in hemoglobin (Hb) level from baseline to day (D) 14 and D28. A total of 92 patients were enrolled and 79 had completed the 28-day follow-up period. The overall cure rate was 98.8% with 95% confidence interval of 0.915–0.998 without polymerase chain reaction correction. The parasite clearance rate was high with fast resolution of clinical symptoms; 100% of the study participants cleared parasitaemia and fever on D3. Gametocyte carriage was reduced from 7% on D0 to 1% on D3 and complete clearance was achieved on D14. Mean Hb concentration significantly increased on D28 compared to that on D14. There was no serious adverse event. AL was efficacious and safe in a high-transmission setting for treatment of uncomplicated *falciparum* malaria.

## Background

Malaria remains a major global public health burden. It is estimated that 214 million cases and 438 000 deaths had occurred due to malaria in 2014 [1]. Climatic and ecological changes, population movements, global warming and expansion of developmental schemes coupled with limitations in existing control interventions are likely to put more people at-risk of malaria than before [2]. Proven malaria preventive strategies currently in use are insecticide-treated nets and indoor residual spraying [3]. However, rapidly developing insecticide-resistant vectors to the available insecticides is jeopardizing the effectiveness of both of these strategies [4]. Larval habitat manipulation and modification, and chemical or biological larviciding have been used as larval source management strategies in certain countries. Despite the success of this strategy to some extent it is not taken as a core strategy to prevent malaria for the reason that a larval habitat should be well-defined for its implementation (reviewed in reference [5]).

Preventive chemotherapy and case treatment are integrated into the above malaria vector control interventions. Early case detection and prompt treatment is the mainstay to minimize malaria-related morbidity and mortality. Currently artemisinin-based combination therapies (ACTs) are the latest and most effective therapies for uncomplicated *Plasmopdium falciparum* malaria [6] which is the most severe and widespread of all human malaria parasites [7]. High efficacy in parasite clearance, rapid resolution of clinical symptoms, and reduced likelihood of developing resistance makes ACTs the drug of choice for the treatment of uncomplicated falciparum malaria [8]. The strong gametocytocidal activity of artemisinin compounds [9–12] is additional benefit to reduce transmissibility and thus further reducing malaria incidence in low-transmission settings.

Artemether-lumefantrine (AL) having trade names *Coartem®*, *Riamet* and *Falcynate-LF* is one of a number of ACTs. In AL the semi-synthetic artemisinin derivative artemether is combined with lumefantrine which is purely synthetic. The drug which is manufactured in tablet form from 20mg artemether and 120mg lumefantrine is currently recommended as the first-line treatment for uncomplicated malaria in several countries [6]. In Ethiopia, AL was introduced as the first-line drug against uncomplicated falciparum malaria in 2004 because of the high treatment failure of sulfadoxine-pyrimethamine [13].

The recent evolution and spread of artemisinin resistant malaria parasite populations in Southeast Asian countries [14, 15] poses yet another threat. Although wide-scale studies that analyzed *P*. *falciparum* isolates from numerous sub-Saharan African countries including Ethiopia found no mutations in the parasite’s K13-propeller gene, which is associated with artemisinin resistance in Southeast Asia [16, 17] the threat is clear. The World Health Organization (WHO) recommends that the efficacy of the first- and second-line antimalarial drugs be regularly assessed for early detection and prevention of spread of resistant parasite populations [18]. For rapid and evidence-based revision of treatment policies malaria drug efficacy studies are vital. Failure to detect the emergence of anti-malarial drug resistance could lead to drug-resistant malaria epidemics with drastic public health and economic consequences. Most malaria epidemics recorded in history were partially attributed to unrecognized resistance to the anti-malarial therapy being used at the time [19].

There are different methods of detecting the emergence and spread of malaria drug resistance *in vivo* therapeutic efficacy study being the gold standard. Knowledge of malaria transmission intensity and factors that influence it is of paramount importance in identifying sentinel sites where antimalarial drug efficacy studies could be conducted [19]. Accordingly, the Ethiopian Federal Ministry of Health (FMoH) has established sentinel sites to conduct national antimalarial drug efficacy studies every two years [20]. A number of studies have already been conducted in different parts of the countryon AL efficacy in largely low malaria transmission, [21–24]. However in Setit Humera, which is one of the sentinel sites and having high malaria transmission, such a study is lacking. This study was, therefore, conducted to assess the therapeutic efficacy of AL against uncomplicated *P*. *falciparum* malaria in Setit Humera Health Center (SHHC).

## Methods

### The study site

Setit Humera is a district town in northwest Ethiopia near the borders with Sudan and Eritrea, some 983 km from Addis Ababa. It is located at 14°17′N latitude and 36°37′E longitude with an altitude of 637 meters above sea level. According to the district agriculture and rural development office record, the town has a total area of 153.03 km^2^ with monthly temperature and rainfall patterns showing great variations. The average annual temperature and rainfall are 27.6°C and 611 mm respectively. Rainfall mostly starts in June peaks in August and stops in the end of September. May is the hottest month with an average temperature of 33.0°C and January is the coldest (24.2°C). Sesame and barley are the main crops in the area. National population projection by the central statistics agency (reported in July 2015) estimated that 30918 people (17801 males, 14768 females) were residing in Humera town [25]. Majority of the residents belonged to the Tigre ethnic group of which many were repatriates from refugee camps in Sudan and others settled from different parts of Tigray Region during resettlement programmes.

The town is situated in Tekezze river basin and thus has suitable micro and macro habitats to support malaria vector mosquitoes. Malaria transmission is seasonal and peaks between mid-September and December as district health office data show. Although rigors epidemiologic studies are lacking the town is characterized as malaria mesoendemic [26–29]. Highlanders having little malaria immunity visit Humera in huge numbers as labor workforce or travelers every year and it is this migrant population which bears the highest malaria burden in the locality. Unpublished reports from the zonal administration office show that close to half a million people influx to Humera during the major labor season every year.

### Study design and population

The study was conducted between October 28, 2014 and January 9, 2015 in SHHC according to the WHO revised protocol for malaria drug therapeutic efficacy study [19]. A minimum sample size of 88 was calculated using the single population proportion formula assuming 5% margin of error, 95% confidence interval (CI), 5% treatment failure and 20% dropout rate [30]. But finally 92 patients were enrolled to increase the statistical power.

Febrile patients (axillary temperature ≥37.5°C) or having history of fever within the previous 24 hours, who fulfilled other inclusion criteria and signed an informed consent were eligible for the study. Specifically, patients of age ≥6 months, body weight >5 kg, microscopically confirmed *P*. *falciparum* mono-infection with asexual parasitaemia of 1000–200,000 asexual parasites/μL of blood, non-pregnant or non-breast-feeding women, permanently living within the health center catchment area (5–10 km radius) during the study period were recruited.

The exclusion criteria were evidence of mixed or mono-infection with plasmodium species other than *P*. *falciparum*, hemoglobin (Hb) level ≤5.0 g/dl, AL intake within the previous 2 weeks, inability to take oral medication or continuous vomiting, known hypersensitivity to AL, severe malaria or other danger signs, severe malnutrition, febrile conditions due to diseases other than malaria (e.g. measles, acute lower respiratory tract infection, severe diarrhoea with dehydration) or other known underlying chronic or severe diseases (e.g. cardiac, renal and hepatic diseases, human immunodeficiency virus (HIV)/acquired immunodeficiency syndrome (AIDS) cases); and regular medication which may interfere with AL pharmacokinetics.

The study was a single-arm prospective evaluation of clinical and parasitological responses to partially supervised treatment of AL for uncomplicated falciparum malaria. Patients who met the inclusion criteria were enrolled and followed-up for 28 days after treatment. The follow-up was based on fixed schedule of check-up visits and corresponding clinical and laboratory examinations. On the basis of the results of these assessments, the patients were classified as having therapeutic failure (early or late) or an adequate parasitological and clinical response. The proportions of patients experiencing therapeutic failure during the follow-up period were used to estimate the efficacy of the study drug.

### Baseline screening

Socio-demographic and clinical data were recorded for each patient. Axillary temperature and body weight were measured. Thick and thin smears were prepared using finger-prick blood samples, and 3–10% Giemsa stained following standard procedure [31]. Malaria parasites were detected, species identified and parasitaemia quantified, and gametocyte carriage determined at baseline and all scheduled and unscheduled visits during the 28-day follow-up period.

Slides were examined by expert microscopists. Asexual parasite density was estimated from thick blood smears by counting the number of asexual parasites against 200 white blood cell (WBC) or against 500 WBC (if the count was <10 parasites/200 WBC) assuming an average WBC count of 8,000/μl blood. A thick blood smear was declared negative if no parasite was detected in 100 high power fields. Gametocytes were detected and counted in a thick film against 500 WBCs. Quality control was conducted on 82 slides by better experienced professional malaria microscopists. Slide preparation and staining quality was rated as “excellent” and “good” respectively.

Hb level was measured from the same finger-prick blood sample on day (D)0, D14 and D28 with a portable spectrophotometer (HemoCue, Ängelhom, Sweden). Anemia was defined according to the WHO classification: Hb<12.0 g/dl for women and children, <13.0 g/dl for adult men; Hb = 10.0–11.9 and 11.0–12.9 g/dl, mildly anemic for children and/or women and adult men respectively; Hb<10 and <11, moderately anemic for children women, and adult men respectively; Hb≤5.0 g/dl was considered severe anaemia and an exclusion criteria [19].

Potential participants were assessed further for adherence to the rest of the inclusion criteria. Baseline physical and clinical examinations with particular attention to any danger signs or symptoms associated with severe malaria were thoroughly assessed by a clinical study team. Febrile patients were treated with an appropriate dose of antipyretics/paracetamol. Patients who met the selection criteria at this stage were assigned patient identification number and referred to the laboratory again. This was to determine the Hb again, repeated thick and thin blood smears preparation, before the patient was enrolled and treated with AL, and for collection of blood spots for late molecular genotyping in case treatment failure >5% occurred. Patients with Hb level <5.0 g/dl and/or positive for mixed infection were excluded from the study and were referred immediately to the outpatient department for an appropriate care. Patients satisfying all the inclusion criteria were enrolled to the study and started partially supervised treatment with AL.

### The follow-up

AL [Batch: DYI473602; Mfg: 08–2013; Exp: 07–2015] which was manufactured by Ipca Laboratories Ltd (Plot №: 255/1; Athal, Silvassa 396 230 (D & NH), India) was provided by the Ethiopian FMoH through WHO support was used. Drug dosage was determined according to the revised WHO weight-based guideline. Accordingly, enrolled patients were treated with the standard six-dose regimen of AL given twice daily for three consecutive days. The first dose of the medication and all morning doses were administered in the health center under direct supervision of the study team. The evening doses of D0, D1 and D2 medications were taken respectively 8 and 12 hours after the administration of the morning dose. Patients and/or parents were instructed how and when to take the evening dose. They were also advised to take fatty meals before or after drug intake to ensure proper absorption of the drug. Success of drug administration at home was checked during the next visit (on D1, D2 and D3).

While the medication given to young children was crushed, mixed with water, and administered as a suspension; to older children and adults tablets were taken orally with a glass of water. Patients were given some peanuts or cream biscuits before/after each dose. At each supervised drug administration the patients were watched for 30 minutes. If vomiting occurred before 30 minutes, the dose was repeated and observed for an additional 30 minutes. Patients vomited more than once were withdrawn from the study and were referred immediately to the outpatient department for rescue treatment with intramuscular or intravenous quinine as per the FMoH guideline.

The evening doses with replacement were given to the patient or caregiver responsible to administer the medications at home with proper and clear verbal instruction when and how to take the medication. They were advised to administer the drugs with some fatty meals. The patients or caregivers were instructed to come back to the health center if the patient had vomited the medication administered at home. One on-duty nurse was assigned to take care of the study patients at the health center at night/off-duty hours.

Patients with admission fever >37.5°C were treated with a standard dose of 10 mg/kg paracetamol tablets every 6 hours until the symptoms subsided. Patients who encountered illnesses other than malaria during the follow-up received standard care and treatment in the health center free of charge. Routine use of non-study medications with antimalarial activity, including antifolate, co-trimoxazole, tetracycline and doxycycline, was avoided. Patients infected with *P*. *vivax* were treated with chloroquine as per the FMoH guideline [32].

On D0 (enrollment day), patients who were successfully treated with the first dose of AL were given an appointment card bearing patient name and identification code and next scheduled visit date, and the evening dose to be administered at home. Patients were then advised to come back for treatment the next two days (D1, D2). Scheduled follow-up visits were on D3, D7, D14, D21, and D28. There were unscheduled visits as well when a participant felt sick.

The participants were interviewed; physically and clinically examined with particular attention to any danger signs or symptoms associated with severe malaria. Participants’ body weights and axillary temperature was measured. Finger-prick blood samples were routinely drawn to prepare blood smears and determine parasitaemia. These routines were done on all the scheduled follow-up days and under emergency visits. Blood Hb level was also measured on D0, D14 and D28.

Patients were excluded from the study after enrollment if D0 repeat parasite count <1000/μL, missed the evening dose on the first three follow-up days, vomited any study dose twice and missed any treatment dose, evidence of severe and complicated malaria, experience of serious adverse events (AEs) that forced discontinuation of treatment, intake of other drugs with antimalarial properties, detection of other malaria species during the follow-up period, development of a febrile illness (e.g. pneumonia, dysentery, measles) that interferes with outcome classification and withdrawal of consent.

### Assessment of AEs

The WHO [19] defines an AE as signs, symptoms or abnormal laboratory finding not presented at enrolment, but occurring during follow-up, or presented at D0 and increased in intensity during follow-up despite clearance of parasitaemia. A serious AE is defined as any adverse experience that resulted in death, life-threatening experience, participant hospitalization, persistent or significant disability or incapacity, or specific medical or surgical intervention to prevent serious outcome.

Evaluations of AE were determined through clinical and physical examination, and questioning with standard list of malaria associated and AL related AEs. Caregivers were asked to inform any unusual phenomena occurred after the administration of the drug including the child’s tolerability to the treatment.

Participants who met the following conditions were excluded from the study at any time. These were withdrawal of consent, failure to complete treatment due to persistent vomiting, failure to attend the scheduled visits including lost follow-up, AE, erroneous inclusion of a patient who did not meet the inclusion criteria; voluntary protocol violation, self or third-party administration of antimalarial drug or antibiotics with antimalarial activity; involuntary protocol violation such as occurrence during follow-up of concomitant disease that would interfere with a clear classification of the treatment outcome; and detection of a mono-infection with another malaria species. Data of withdrawn patients were censored during analysis and reasons for discontinuation or protocol violation were recorded.

### Treatment outcomes (endpoints)

Study endpoints were classified into primary and secondary endpoints. The primary outcome was the D28 overall efficacy of AL expressed as polymerase chain reaction (PCR)-uncorrected cumulative success rate (or the cumulative failure rate) and the proportion of adequate clinical and parasitological response (ACPR). Or it is the proportion of early treatment failure (ETF), late clinical failure (LCF) or late parasitological failure (LPF), or withdrawal. The primary outcome was analyzed using the Kaplan-Meier (K-M) survival estimator and standard WHO per-protocol (PP) analysis.

The secondary outcomes were parasite clearance rate (proportion of patients with negative blood smears on D1, D2 and D3), fever clearance rate (proportion of patients without fever on D1, D2 and D3), gametocyte carriage rate (proportion of patients with gametocytes during the 28 day follow-up period), hematological recovery (change in mean blood Hb concentration from baseline to D14 and D28). Details of each treatment endpoint are found in WHO 2009 [19].

### Data management and monitoring

An enrollment and a case record forms were completed for each study subject. Patient name initials and study code on the case record form were used to identify individuals. The first author was responsible to complete the case record forms at each visit. All corrections were made on the case record form by striking through the incorrect entry with a single line and entering the correct information adjacent to it.

All clinical data were recorded in standardized case record forms. Laboratory data were recorded in a laboratory record book and then transferred to the case record forms. Data were transferred from the case record forms into a computerized database (IBM SPSS, WHO excel sheet and plain excel spread sheet). The WHO excel sheet is specially designed for the analysis of efficacy study data and it only performs the analysis when double entry is assured to verify accuracy of entry. Two backup files and the database were stored on compact discs after each data entry session.

Members of the central research team from the Ethiopian public health institute (EPHI) supervised the overall progress of the study and reviewed all data records at the mid of the study period. All record forms were checked for completeness and accuracy. In addition, a daily communication was made with the members of the central team.

### Data analysis

Data were double entered into the WHO Excel spread sheet designed for therapeutic efficacy data. Data were also entered into IBM SPSS (version 20) software to calculate descriptive statistics (mean, standard deviations, percentages). Independent sample t-test was used to compare baseline temperature and parasitaemia; D1 body temperature between children and adults; mean blood Hb level at D0, D14 and D28 between patients with parasitaemia ≥10,000 and <10,000/**μL** blood. The paired sample t-test was used to to compare D1 parasitaemia between children and adults and and to compare mean Hb level between D0 and D14, D0 and D28, D14 and D28. All comparisons were performed at 95% CI and significance level of 0.05.

### Ethical considerations

The study protocol was approved by College of Natural Sciences Institutional Ethics Review Board, Addis Ababa University. Written informed consent was obtained from adult participants. Parents/caregivers gave their consent for children. Free medical services were provided to the participants during the follow-up. Participant round-trip transport cost was also covered during every scheduled visit. Participant identities were kept confidential throughout.

## Results

### Study population

A total of 1878 malaria suspects attending SHHC during the study period were screened. Of these, 1258(67%) were malaria slide-positive with 986(78.4%) *P*. *falciparum*, 253(20.1%) *P*. *vivax* and 19(1.5%) mixed *P*. *falciparum/P*. *vivax* infections. Most of these malaria patients were mobile laborers from diverse parts of Ethiopia and hence were not potential candidates for inclusion in the AL efficacy study. Only 120 were found eligible and targeted. Of these; 18 refused consent, 4 had parasitaemia <1000/μL, 4 were lactating and 2 pregnant women.

Thus, only 92 patients finally satisfied the inclusion criteria and enrolled. At baseline, 57(61.9%) patients were males yielding 1.63 male to female ratio (Table 1). Three (3.3%) were under-five (U5) children, 33(35.9%) 5–14 years old, and the remaining 56 (60.9%) were over 14 (adults). Mean body temperature was 38.5±1.25°C. While 61 patients (66.3%) were febrile; the rest had self-reported history of fever within the previous 24 hours. The mean body temperatures of children and adults were 38.3±1.20 and 38.8±1.27, respectively, with no significant difference. The mean body weight was 39.6±14.13.

**Table 1:** Baseline characteristics of the study population.

Characteristics	Age category (year)			
	U5 (N = 3)	5–14 (N = 33)	>14 (N = 56)	Total (N = 92)
Mean age (or range)	3(2–4)	9.73(5–14)	18.96(15–28)	15.13(2–28)
Males (no(%)	3(3.3)	22(23.9)	32(34.8)	57(61.9)
Females (no(%)	0(0.0)	11(12.0%)	24(26.1)	35(38.1)
Mean body temperature (°C)±SD	39.3±1.37	38.7±1.20	38.4±1.27	38.5± 1.25
Mean body weight (Kg)±SD	11.3±2.30	26.4±8.67	48.9±7.10	39.6±14.13
Mean Hb (g/dL)±SD	11.4±1.05	12.3±1.52	13.8±1.74	13.2±1.82
Mean parasitaemia (per μL) ±SD	28846.67±37523	30389±42421	26215±34642	27798±35942
Gametocyte carriage (%)	1(1.1%)	3(3.3%)	3(3.3)	7(7.6%)

SD: standard deviation, N = baseline number, Kg: kilogram, Hb: hemoglobin, no: number

Baseline mean Hb level was 13.2±1.83 and 26 patients (28.3%), 17 children (below 14 years of age) and 9 adults, were anemic (5 moderate and 21 mild). Overall baseline mean parasitaemia was 27798(±35942)). There was no significant variation between mean parasitaemia for children (30259±41518) and those over 14 years of age (26215±34642). There were 6 patients with parasitaemia >100,000/μl. Overall, 16 of the 26 anemic patients were males. Gametocyte carriage rate was 7.6% with gametocyte number ranging from 48-2224/μL of blood. Most study participants (82.6%) reported previous history of clinical malaria and 95% of the participants/parents confirmed the availability of at least one bed net per household.

**Fig 1:**
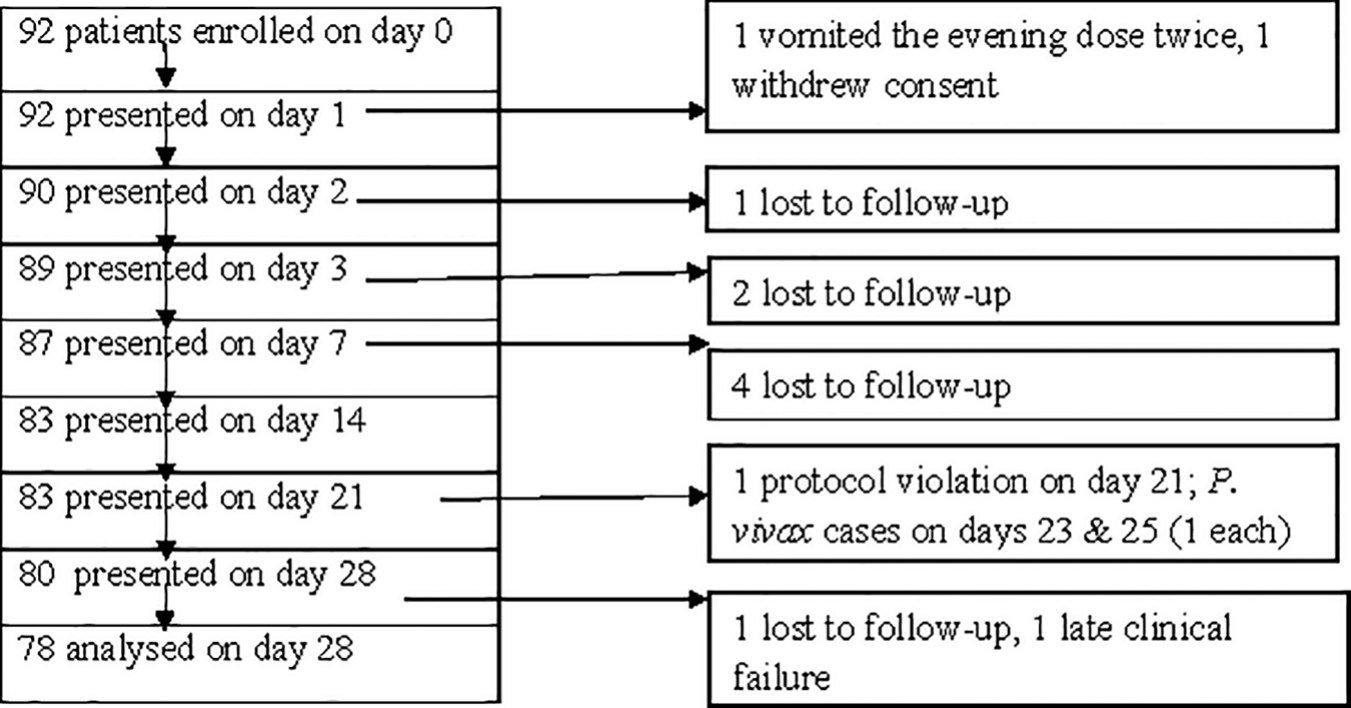
Enrolled and excluded study participants during the 28-day follow-up.

Among the 92 participants, 13 exclusions were recorded on different follow-up days for various reasons. Two were excluded on D1 (1 repeated vomiting of the evening dose, 1 withdrawal of consent); 1 on D3, 2 D7, 4 D14 and 1 on D28 all due to LFU; 1 on D23 and 1 on D25 both had *P*. *vivax* infection; and 1 on D21 because of intake of non-study antimalarial drug or protocol violation (Fig 1).

### Primary outcome

The cure rate was 98.7% based on PP analysis method with 1 LCF (parasitaemia 9200/**μ**L on D28) and no ETF or LPF (Table 2). PCR-uncorrected cure rate was 98.8% (95% CI: 0.915–0.998) as the K-M survival estimate analysis showed (Table 3).

**Table 2:** Summary of treatment outcomes based on PP analysis stratified by age.

Age (year)	U5 (N = 3)	5–14 (N = 33)	>14 (N = 56)	Total (N = 92)
	n(%)	n(%)	n(%)	n(%)
ACPR	3(100)	30/31(96.8)	45(100)	78(98.7)
ETF	0(0.0)	0(0.0)	0(0.0)	0(0.0)
LCF	0(0.0)	1(3.1)	0(0.0)	1(1.3)
LFU	0(0.0)	0(0.0)	8(14.3)	8(8.6)
LPF	0(0.0)	0(0.0)	0(0.0)	0(0.0)
W	0(0.0)	2(6.1)	3(5.4)	5(5.4)

ACPR: adequate clinical and parasitological response, ETF: early treatment failure, LCF: late clinical failure, LFU: lost follow-up, LPF: late parasitological failure, W: withdrawal, U5: under-five, N: baseline number, n: final number, %: percent

**Table 3:** Summary of PCR-uncorrected cure rate based on Kaplan-Meier survival analysis.

Follow-up day	n	Failure	Censored	Success cumulative incidence	Failure cumulative incidence
0	92	0	0	1.000	0.000
1	92	0	2	1.000	0.000
2	90	0	1	1.000	0.000
3	89	0	2	1.000	0.000
7	87	0	4	1.000	0.000
14	83	0	0	1.000	0.000
21	83	0	1	1.000	0.000
23	82	0	1	1.000	0.000
25	81	0	1	1.000	0.000
28	80	1	1	0.988	0.013
Total	78	1	13	95% CI: 0.915–0.998	95% CI: 0.002–0.085

### Secondary outcomes

**Fig 2:**
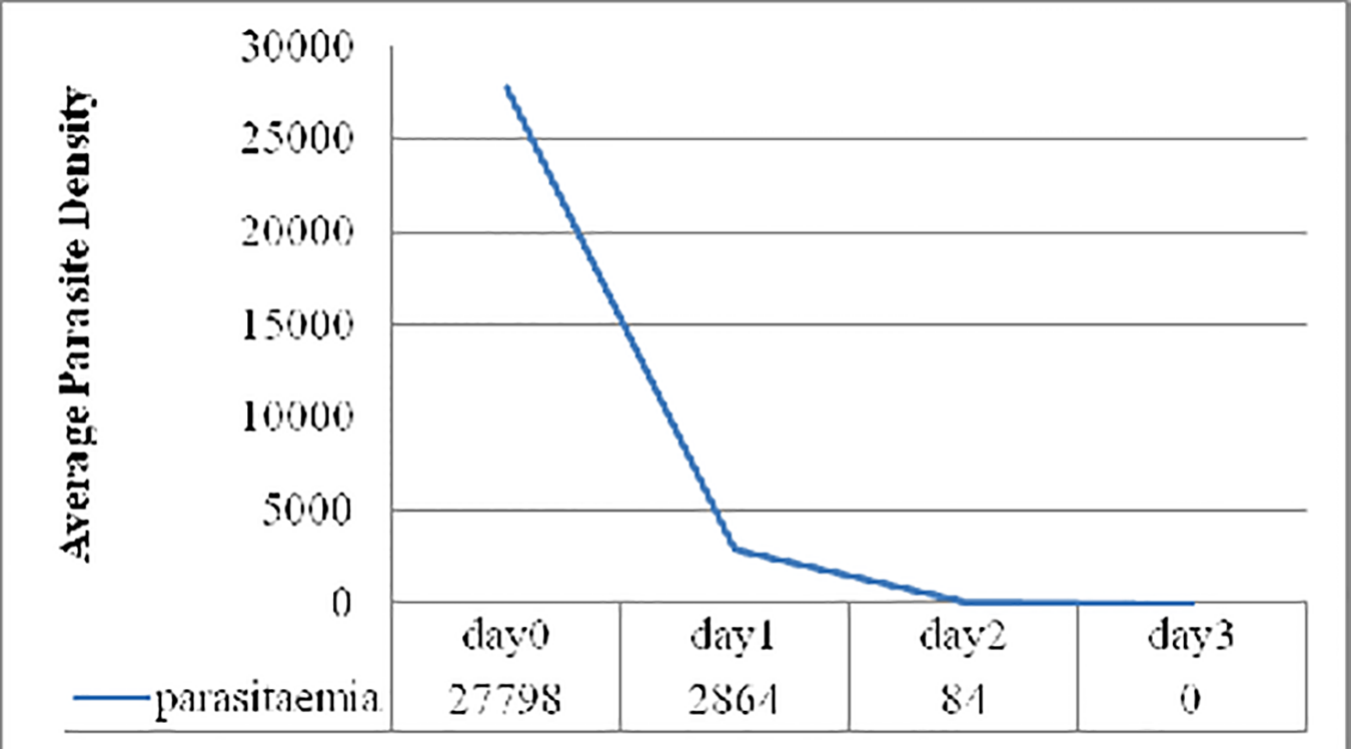
Graphic presentation of mean parasite density on the first three follow-up days.

Parasite clearance rate was high that 33% of the patients cleared parasitaemia on D1, 84.4% on D2 and 100% on D3. The mean parasitaemia declined from 27798 on D0 (baseline) to 2864 on D1, 84 on D2 and to 0 on D3 (Fig 2). Taking D2 mean parasitaemia as a comparing variable, parasite clearance rate was compared between children and adults and there was no significant difference.

**Fig 3:**
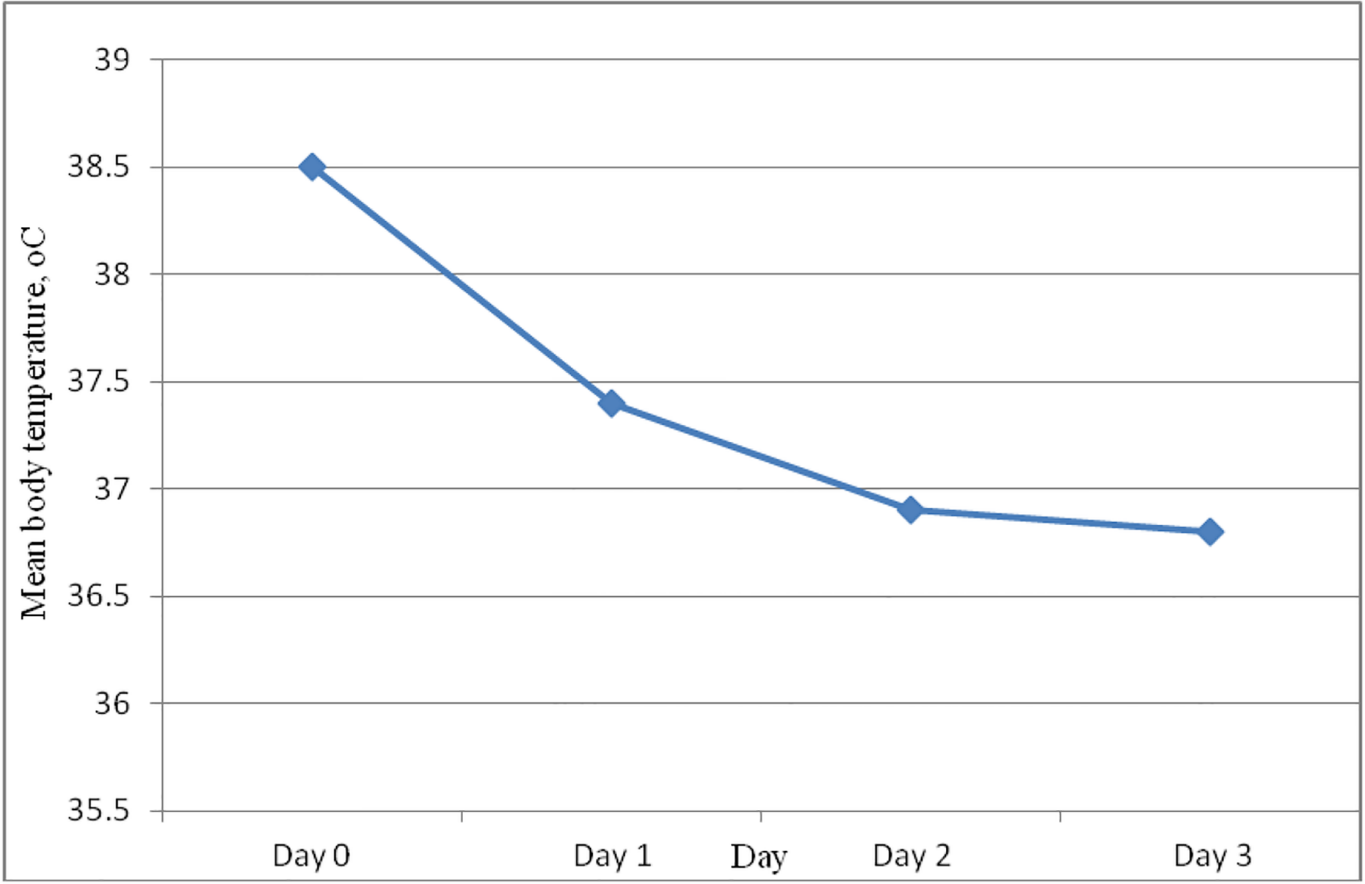
Mean body temperature on the first three follow-up days.

At baseline, all study participants were considered febrile; 79 of them had actual fever, while the remaining 13 had self-reported fever within 24 hours before enrollment. The mean body temperature declined from 38.5°C on D0 to 37.4°C on D1, 36.9°C on D2 and to 36.8°C on D3 (Fig 3) with 80% of the participants clearing fever on D1, 97.8% on D2 and 100% on D3. Fever clearance rate was compared between children and adults taking D1 mean temperature as comparison variable; D1 mean body temperature was significantly higher (p = 0.022) in children (37.7±0.90) compared to adults (37.4±0.67) implying adults cleared fever faster than children.

**Fig 4:**
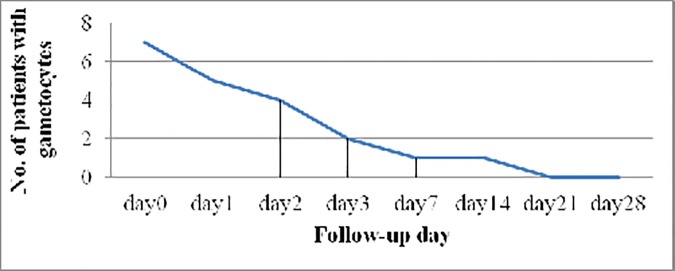
Number of participants with gametocytes on each follow-up day.

The number of gametocyte carriers had declined from 7 on D0 to 5, 4 and 2 on D1, D2 and D3 respectively; only one patient remained gametocyte carrier on D7 and D14 and no gametocytes were detected beyond D14 (Fig 4).

The number of anemic patients which was 26 at baseline had surprisingly risen to 42 (20 adults, 22 children) and 30 (18 adults, 12 children) on D14 and D28 respectively. The mean Hb level did not increase during the follow-up period compared to the baseline (D0). Rather it appeared to significantly decrease (p<0.0001) between D0 (13.2±1.82) and D14 (12.2±1.64). Similarly, D28 mean Hb level (12.8±1.42) was significantly lower (p = 0.003) than that on D0. However, there was a significant rise in mean Hb level on D28 (12.8±1.42) compared to D14 (p = 0.002). Comparison of D0, D14 and D28 mean Hb level between patients with parasitaemia ≥10,000 and <10,000/μL showed no significant difference (13.0±1.88, 13.6±1.71, p = 0.136; 12.0±1.66, 12.36±1.67, p = 0.403; 12.8±1.50, 12.8±1.32, p = 0.845 for ≥10,000 and <10,000/μL; and for D0, D14 and D28 respectively.

### Incidence of AEs

At baseline, a number of signs and symptoms were either self-reported or witnessed (Table 4). Headache was most frequent being reported by 8 patients followed by perioral alcer (7), anorexia (6), diarrhea (3), vomiting (2), cough (6), abdominal pain (5), dizziness and nausea (4), weakness/fatigue (4), and sleep disorder (5). Most of these probable AEs disappeared with the clearance of parasitaemia except cough and perioral ulcer. Whereas cough persisted for sometime beyond parasite clearance, perioral ulcer was noted after clinical manifestations subsided.

**Table 4:** Probable drug-related AEs observed during the course of the study (N = 92).

Description	Frequency	Percent
Headache	8	8.6
Perioral ulcer	7	7.6
Anorexia	6	6.5
Cough	6	6.5
Sleep disorder	5	5.4
Abdominal disorder	5	5.4
Dizziness	4	4.3
Weakness/fatigue	4	4.3
Diarrhea	3	3.4
Vomiting	2	2.2
Total	50	54.9

## Discussion

The 67% malaria prevalence indicates the heavy burden of malaria in the study area calling for scaling-up of control interventions and identifying possible local risk factors. Most previous AL efficacy studies in Ethiopia were in low transmission areas where malaria prevalence rates were much lower: 26.3% in Kersa, South West Ethiopia [21], 21.1% in Bahir Dar district, Northwest Ethiopia [22] and 21.7% in Oromia State, Central Ethiopia [33]. The 98.8% PCR-uncorrected cure rate shows the high therapeutic efficacy of AL some nine years after its introduction for the treatment of uncomplicated falciparum malaria in the study site which is a high transmission setting. Although this result is slightly lower compared to the drug’s baseline efficacy (100%) in the area [34] it is relatively higher than PCR-corrected AL cure rates [96.3–97.8%] documented in other parts of Ethiopia [21, 35–36]. A PCR-uncorrected cure rate of 98.8% was documented in a nearby malaria endemic area along the Ethio-Sudanese border [37]. A study by Nega and colleagues conducted in central eastern Ethiopia recorded two treatment failures having PCR-uncorrected failure rate of 2.4% (2/83). However, only one of the two cases was confirmed to be a recrudescence giving a failure rate of 1.2% when genotyped [38]. The study period of both of those studies was the same as the present one.

The absence of ETF confirms nonexistence of possible artemisinin-resistant *P*. *falciparum* strains in the study area. Detecting parasitaemia on D3 is key indicator to suspect artemisinin resistance [39]. The absence of ETF and low recurrent malaria (only 1 LCF) in the present study could imply high therapeutic efficacy of both components of AL in the study site. Studies in Ethiopia [23] and Nigeria [40] reported two and three ETF cases of AL treatment respectively.

Factors such as host nutritional and immune status, initial parasitaemia level, pharmacokinetics and pharmacodynamics may influence the therapeutic efficacy of a drug apart from inherent parasite susceptibility [19]. These and other related factors may result in treatment failure showing low efficacy of otherwise an efficacious drug. At the same time, resistant parasites may be cleared with the help of the immune system which may result in exaggerated efficacy of otherwise a less efficacious antimalarial drug. As a result, the WHO recommends the integration of pharmacological studies as standard protocol of therapeutic efficacy studies to increase the determining capacity of therapeutic efficacy studies [19].

In this study, the baseline mean parasitaemia was 27798 (±35942). Parasitaemia is linked to the degree of malaria severity and hence is an important parameter to help in the decision of the type of treatment to be initiated. It has also epidemiological implications as it indicates the level of transmission intensity in a specific area. Although the level of malaria endemicity in the area is not thoroughly determined Mengesha and coworkers [28] characterized it as mesoendemic. According to local health office data, malaria is unstable and seasonal with varying duration and intensity mainly depending on the amount and length of rainfall. Accordingly, the level of transmission was high during the study period as rainfall amount was high with extended duration.

Clearance of parasitaemia was achieved by all patients on D3 indicating high parasite clearance rate. This is in line with the established quality of AL, rapid clearance of parasitaemia. This could be due to the inherent capacity of artemether to rapidly metabolize to its active ingredient, dihydroartemisinin, and rapidly absorbed to the bloodstream which results in rapid clearance of parasite biomass [35]. The rate of elimination of artemether is also fast with a half-life time ranging from 2–3 hours [41]. The partner drug, lumefantrine, is a slowly acting drug with long half-life time ranging from 4–7 days [42]. This leads to successive accumulation of the drug after the completion of the full dose sufficient enough for the elimination of residual parasites and possibly prevention of new infections. The high parasite clearance rate of AL is also indicated in other efficacy studies conducted in Ethiopia [23, 36]. Slower parasite clearance rate was associated with AL resistance in the Great Mekong region [15, 43].

In Eritrea, artesunate and amodiaquine (AS+AQ) is the first-line treatment of uncomplicated falciparum malaria and oral quinine is the second-line treatment [1]. So comparison of AL efficacy in Ethiopia with its northern neighbor is not possible. While artesunate + sulphadoxine/pyrimethamine (AS+SP) is the first-line drug AL is the second-line against uncomplicated falciparum malaria in Sudan. A recent large trial report from the country revealed a declining efficacy for the former and the latter was found to be highly efficacious [44]. Although AS+SP remains the first-line drug for the treatment of uncomplicated falciparum malaria in Yemen as well, AL showed higher efficacy than the former in the clearance of gametocytaemia [45]. The therapeutic efficacy of AS+SP and AL was evaluated in Somalia in 2013 and 2015 for the treatment of uncomplicated falciparum malaria and it was found that AS+SP (the first-line drug) failure rate was above the threshold (10%) for policy change. In contrast, AL was reported highly efficacious and was selected to replace AS+SP as the first-line treatment in 2016 [46]. In a recent multicentre trial done in six research centers across Tanzania, Burkina Faso and Kenya AL efficacy was similarly high for the treatment of uncomplicated malaria in children [47].

AL treatment failures among European travelers returning from African countries such as Sierra Leon [48], Congo [49] and Tanzania [50] have been reported. On the other hand, failure of dihydroartemisinin-piperaquine (DHA-PPQ) was reported for the treatment of *P*. *falciparum* malaria in an Italian tourist returned from Ethiopia [51]. Post-treatment prophylactic effect of DHA-PPQ against re-infections is considered longer than that of AL [52]. In Ethiopia DHA-PPQ failures have not been reported before. DHA-PPQ-resistant strain of *P*. *falciparum* originating in Equatorial Guinea was also identified more recently in a patient in China [53].

Fever is the main clinical manifestation of malaria causing much discomfort. In this study, the baseline mean body temperature was 38.5±1.25°C and all participants were considered febrile. There was no significant difference in baseline mean body temperature between children and adults. This may be explained by the mesoendemic endemicity level, seasonal and unstable nature of malaria transmission in the study site. It is well known that partial immunity is not acquired in areas with unstable and seasonal transmission pattern. The fever clearance rate was rapid, notably less than three days. The fast fever resolving capacity of AL is also observed in other efficacy studies conducted in Ethiopia [21, 35, 54] as well as elsewhere in sub-Saharan Africa [55, 56] as opposed to findings from Southeast Asian countries [15,43]. Like a delay in parasite clearance, fever clearance rate is considered the main phenotypic presentation of artemisinin-resistance in Southeast Asia.

The high parasite and fever clearance rates could be explained by the fast act of artemether to clear parasite biomass leading to rapid resolution of clinical manifestations [6]. Fever clearance rate was higher in adults than children. This probably suggests the development and maintenance of malaria immunity, to some extent, in adults although both groups are equally vulnerable to clinical malaria given the epidemiological context of the setting.

The baseline mean Hb level was significantly higher than that on both D14 and D28. This is contrary to what was expected. Hb level is expected to increase or at least remain unchanged following parasite clearance. Results of other studies from Ethiopia and other African countries showed slight to significant increase in mean Hb level following treatment with AL [23, 54, 56]. Since the etiology of anemia is multiple in Africa non-malaria anemia may be suspected in this case. Apart from that, it is harder to explain this observation. Indeed Gray and colleagues [57] reported anemia as severe AE of AL treatment. As reviewed in Rehman and others [58], late hemolysis leading to significant decrease of Hb was evidently associated with all artemisinin derivatives and all routes of administration (intravenous, intra-muscular, intra-rectal and even oral).

The probable AEs observed in this study were almost similar with observations of other studies in Ethiopia [22, 34, 54, 59]. Most of the possible AEs reported from study participants and/or observed during physical and clinical examination were similar to the symptoms of malaria itself.

However, cough persisted longer (up to D14) after other symptoms subsided. So we suspect it as a drug related AE. Further study is required to exactly determine whether it is drug related or due to other concomitant upper respiratory tract infections that cause chronic cough. Similarly, peri-oral ulcer (not a common malaria symptom) was observed in 7 patients similarly after the resolution of other malaria related clinical manifestations. A similar report is found in other efficacy studies [21, 33, 60]. It is therefore, very unlikely to consider this occurrence to be purely due to chance.

The result of this study showed strong gametocytocidal effect of AL. It reduced the gametocyte carriage from 7 (7.6%) on D0 to 1 (1.1%) on D7 and D14 achieving complete clearance on D21. This result is consistent with other studies in Ethiopia and elsewhere [55, 59, 61]. Two separate studies conducted in Ethiopia showed higher gametocyte clearance rate (all cleared at D7) [23, 60] although in another study conducted in Southern Ethiopia gametocytes persisted up to D28 [35].

In areas of high endemicity recurrent infections are common although only a single case was found during the follow-up in the current study. A study in Tanzania, with high *P*. *falciparum* transmission found similarly high AL efficacy [62]. However, the authors reported that almost half of the patients had recurrent infections during follow-up and PCR analysis showed that nearly all those infections were re-infections. A previous report from Uganda in a comparable setting [63] found a similar result. A concern with ACTs in high-transmission areas is that the slow clearance of the partner drugs may facilitate selection of resistant parasite strains [64–66]. In the above same Tanzanian study genotypes associated with tolerance and resistance to lumefantrine were selected necessitating a closer future surveillance of AL efficacy and effectiveness in Africa.

The limitation of this study is not using PCR to distinguish between recrudescence and re-infection. The status of the single LCF was not determined.

## Conclusions

The study found high therapeutic efficacy of AL for treatment of uncomplicated falciparum malaria with high parasite clearance rate and rapid resolution of fever. AL was also effective against the transmissible sexual phase of the parasite (the gametocytes).

## Abbreviations

ACTs: artemisinin-based combination therapies
ACPR: adequate clinical and parasitological response
AEs: adverse events
AL: Artemether-lumefantrine
CI: confidence interval
ETF: early treatment failure
EPHI: Ethiopian public health institute
K-M: Kaplan-Meier
LCR: late clinical failure
LPF: late parasitological failure
PP: per-protocol
PCR: Polymerase Chain Reaction
SHHC: Setit Humera Health Center
WBC: white blood cell
WHO: World Health Organization
